# Mental health planning at a very early stage of the COVID-19 crisis: a systematic review of online international strategies and recommendations

**DOI:** 10.1186/s12888-020-03015-y

**Published:** 2021-01-15

**Authors:** Nerea Almeda, Carlos García-Alonso, Luis Salvador-Carulla

**Affiliations:** 1 Department of Psychology, Universidad Loyola Andalucía, Seville, Spain; 2 Department of Quantitative Methods, Universidad Loyola Andalucía, Seville, Spain; 3 Centre for Mental Health Research, Research School of Population Health, ANU College of Health and Medicine, Australian National University, Canberra, Australia

**Keywords:** COVID-19, Strategies, Recommendations, Mental health, Systematic review

## Abstract

**Background:**

Mental health care systems have been dramatically affected by COVID-19. Containment measures have been imposed, with negative consequences on population mental health. Therefore, an increase in both symptomatology and mental disorder incidence is expected. This research aims to identify, describe and assess the empirical background on online strategies and recommendations developed by international organizations and governments to cope with the psychological impact of COVID-19 at a very early stage of the pandemic.

**Methods:**

The PRISMA guidelines were adapted to review online documents. A new questionnaire was developed to identify the existence of common patterns in the selected documents. Questions were classified into three domains: COVID-19 information, mental health strategies and mental health recommendations. A two-step cluster analysis was carried out to highlight underlying behaviours in the data (patterns). The results are shown as spider graphs (pattern profiles) and conceptual maps (multidimensional links between questions).

**Results:**

Twenty-six documents were included in the review. The questionnaire analysed document complexity and identified their common key mental health characteristics (i.e., does the respondent have the tools for dealing with stress, depression and anxiety?). Cluster analysis highlighted patterns from the questionnaire domains. Strong relationships between questions were identified, such as *psychological tips for maintaining good mental health and coping with COVID-19* (question n° 4), *describing some psychological skills to help people cope with anxiety and worry about COVID-19* (question n° 6) and *promoting social connection at home* (question n° 8).

**Conclusions:**

When fast results are needed to develop health strategies and policies, rapid reviews associated with statistical and graphical methods are essential. The results obtained from the proposed analytical procedure can be relevant to a) classify documents according to their complexity in structuring the information provided on how to cope with the psychological impact of COVID-19, b) develop new documents according to specific objectives matching population needs, c) improve document design to face unforeseen events, and d) adapt new documents to local situations. In this framework, the relevance of adapting e-mental health procedures to community mental health care model principles was highlighted, although some problems related to the digital gap must be considered.

## Background

The COVID-19 outbreak has created an unprecedented crisis in modern health care systems [1]. This disease originated in Wuhan (Hubei Province, China) at the end of 2019 [2]. On January 30th, 2020, the World Health Organization [3] (WHO) declared a public and international health emergency.

On May 1st, 2020, there were 3,318,428 confirmed cases and 234,250 deaths worldwide [4]. The incidence and mortality rate of COVID-19 have saturated health systems in a number of countries. To a greater or lesser extent, containment measures such as social distancing, quarantine and self-isolation have been imposed worldwide [5]. Containment measures can have negative impacts on population mental health (MH) [6]. People may be affected during and after containment by the precipitation of new symptoms or aggravation of previous conditions [7–10]. Considering the vulnerability of MH users, decision makers should also prevent a potential increase in inequalities because MH users experience more distress, fear, anxiety and depression than the general population [11], which can worsen their previous circumstances [12].

Female gender, being a student and poor health status are factors that have been associated with greater psychological impact [13]. Li, Wang, Xue, Zhao and Zhu [14] found that after the COVID-19 outbreak, negative emotions increased, while happiness and life satisfaction decreased. Patients with COVID-19 manifest posttraumatic stress symptoms before and after discharge [15, 16]. People in quarantine and COVID-19 patients feel fear, boredom, anger, loneliness and/or stress [17] and may suffer from hate crimes, fear, alienation and discrimination [18]. People who have lost their loved ones are prone to developing MH complications related to grief after their unexpected loss and social isolation [19].

Professionals who care for COVID-19 patients are highly susceptible to psychological burden [20]. The fear of becoming infected, the suffering of patients and relatives, shortages of medical and protection supplies, and socioeconomic uncertainty increase their psychological impact [21, 22]. Therefore, it is crucial to support these professionals by designing specific MH interventions [23], such as developing screenings to detect depression and anxiety [17] and to assess the psychological condition of COVID-19 patients [7].

In this new environment, it is likely that the balanced MH care model [24] must be adapted to consider the problem of dealing with the community-based care paradigm in a situation where a safe distance between people may be mandatory. For inpatient care, the scientific literature recommends screening users for COVID-19 symptomatology before and during hospitalization to reduce visits and to reduce the size of psychotherapy groups to minimize nonessential contacts [25]. Patients should be isolated in rooms and the length of stay should be reduced to minimize hospital-acquired infection risks [26]. Regarding community treatment services (partial hospitalization, intensive outpatient care, day care and medication services), telepsychiatry is highly recommended to limit contact, except in cases of life-threatening emergencies [26].

The Italian Society of Epidemiological Psychiatry [27] described a proposal for MH care provision during the pandemic: 1) outpatient care should be mainly provided remotely, by telephone or videoconference; 2) in case of crisis, users could access acute MH care in hospitals; 3) day care services will be closed during the pandemic, but necessary online interventions such as telephone monitoring and face-to-face home visits should be available; 4) patients in clinical emergencies will be admitted to inpatient units, although the length of stay should be kept to a minimum and, whenever possible, the crisis should be treated outside of the hospital; and 5) short- and long-term residential care facilities will provide care normally but without admitting new patients, except as an alternative to inpatient care. Special prevention measures, such as training in the use of personal protection equipment, must be available at any time or place.

The Australian government has developed a set of digital resources based on phone and online support services, including counselling, digital peer support and emergency lines [28], in addition to the *Better Access Pandemic Support* resource for providing MH care [29]. Users have access to psychiatrists, psychologists and general practitioners; receive 10 initial individual sessions and, when needed, can receive 10 additional sessions.

In Canada, the Centre for Addiction and Mental Health [30] has developed a long-term system-wide response, including MH resources, care, new models of MH care provision, workplace MH, investment in social determinants of health and a public health approximation for alcohol policy. This strategy highlights the virtual MH care provision trying to guarantee continuity of care and meet user needs (including information) while maintaining physical distancing.

The United States has developed a specific set of recommendations for each main type of care [31]: (a) virtual and telephone-based MH for outpatient care, (b) screening tests for partial hospital programmes, (c) screening protocols, triage for risk exposure, rapid tests before admission and physical distancing in emergency psychiatric care, (d) special inpatient care units for people infected with COVID-19, (e) virtual care for consultation liaison services and staff protection with PPE if virtual care is not possible, and finally, (f) secure response protocols for shelter homes and mobile care at home for community care services.

The Royal College of Nursing and the Royal College of Psychiatrists in the United Kingdom have developed *COVID-19 guidance on mental health care delivery* [32]. In this framework, an inpatient care routine review is recommended to avoid close contact activities and reduce their duration, maintaining attendance quality [33]. In psychiatric intensive care, it is recommended to balance benefits and risks prior to patient admission, provide information about the risks, carry out control measures and isolate patients in case of infection, among other actions [34]. Regarding outpatient care, it is crucial to provide continuity of care (including specific interventions to reduce crises), ensure medication continuity and keep patients and families informed [35]. Finally, for residential care facilities and supported living services, safe working measures are required by developing isolation precautions and increasing the utilization of telepsychiatry [35].

Face-to-face interventions have to be restructured in this pandemic [7]. e-MH should be reinforced [36] because it increases accessibility and equity but only when MH staff are specifically trained [37, 38]. In this framework, telepsychiatry has achieved a relevant role in providing MH care, especially in prevention, psychoeducation, counselling, treatment and emergency interventions [39]. However, this model shows some drawbacks, especially for older adults who lack digital skills [40] and people with cognitive impairments.

On the other hand, general prevention strategies should be based on family support, education and self-care, where interdisciplinary agencies (health, housing, employment and education) should collaborate to identify risk factors and promote help-seeking [41].

Several international organizations have published many recommendations and tips to cope with this health crisis. The WHO proposed to guide people in psychosocial aspects related to COVID-19, ensure referral pathways among sectors, provide MH and psychosocial support for all people, and meet population needs in people with pre-existing conditions, older adults, etc. [42]. In addition, the WHO is concerned with avoiding stigma and addressing bereavement and other psychological consequences. The American Psychological Association (APA) [43] provides a wide range of resources for e-MH and telepsychology. It shares scientific findings that can help people cope with the emotional impact of the pandemic. For the general population, specific strategies have been proposed for reducing stress, anxiety and grief, including measures for those collectives triggered by domestic violence and child abuse. The APA advises seeking help when needed, so it is necessary to improve accessibility to MH services. Staying healthy at home is essential for the United Nations (UN) [44], especially for those suffering from anxiety or stress. The UN’s MH strategy proposes good practices for teleworking, wellbeing management and talking with children; highlighting the roles of telepsychiatry and telecounselling. The UN is concerned with the existence of reliable information sources on how to access MH services and obtain medication. Globally, strategies to mitigate the pandemic impact focus on promoting engagement with MH users, clinicians and state policies [45].

This research aims to identify, describe and assess the empirical background on online strategies and recommendations developed by international organizations and governments to cope with the psychological impact of COVID-19 at a very early stage of the pandemic. Potential common patterns (links between questions) among selected documents will be identified and described.

## Methods

This section has been divided into the following analytical processes: a) methods for study selection (two items: *search strategy and eligibility criteria* and *study selection*), b) questionnaire design, c) variable grouping, d) data collection procedure and, finally, e) cluster analysis of the indicator groups.

### Search strategy and eligibility criteria

In this paper, a strategy is a set of general orientations to guide the design and development of specific policies. A recommendation is a specific action—clinical or organizational—implemented by decision makers to change the situation of any system (in this case, an MH system).

Due to the urgent character of current policy-making, an adaptation of the PRISMA guidelines [46] was developed to carry out the systematic review. The search strategy was based on the PICOS research question where the population (P) was MH services and systems (“mental health service*” OR “mental health system*”). The intervention (I) was any international online MH strategy or recommendation developed to address the COVID-19 psychological impact (“strateg*” OR “recommendation*” AND “Covid-19” OR “2019-nCOV” OR “SARS-COV-2”). The comparator (C) was not applicable. The outcomes (O) were any online international report or guide that included any MH and/or psychosocial considerations for the virus (“report*” OR “document* OR “guideline*). Finally, setting (S) referred to the countries included in the review. Although the PRISMA guidelines were initially designed for reviewing research articles where their (P) was “patients”, previous work has shown that the methodology is also appropriate for reviewing studies whose population or unit of study are MH services or systems [47–49].

The initial search strategy was “COVID” + “mental health”. This retrieved a huge number of records (millions) that could not be managed. For this reason, new constraints were added to the initial search strategy. After checking different key word combinations, the final search strategy comprised the main terms (translated to the corresponding languages): “mental health service*” OR “mental health system” AND “strateg* OR “recommendation* AND “COVID-19” AND “report” OR “document” OR “guideline”. This search strategy and the corresponding inclusion criteria were checked by two panels of experts (including psychiatrists, psychologists, managers and policy makers), one from the International CIty and urban Regional CoLlaborativE (I-CIRCLE) group and the other from the PSICOST research group.

The search strategy was piloted in Google on April 15th, 2020. To keep Google variability under control, two authors (NA and CGA) piloted the search strategy in different computers and places (geographical areas). The Google platform was selected because it provides access to government, institution, association and mass media webpages where target strategies and recommendations, which are not included in the standard indexed databases, are published.

The inclusion criteria were online MH reports or guides published by governments, associations, institutions and relevant media at any time to address the psychological impact of COVID-19. The languages included were English, Spanish, French, Italian and Portuguese. Countries included in the review were those that had developed strategies and recommendations before 15 April 2020 and were accessible from Google. These countries were Australia, Canada, China, England, Finland, Germany, Switzerland and France, Greece, Ireland, Italy, Mexico, New Zealand, Portugal, Spain, Scotland and the United States of America.

The reasons for exclusion were as follows: a) documents not focused on general MH, b) lacking information about online MH care provision, c) including strategies for workers and d) studying the pandemic economic impact.

### Study selection

CGA and NA performed the selection process in the eligibility phase by reading the document full text, including their links. The concordance rate between authors was analysed using Kappa and two-way random ICC tests. Selected documents were characterized by including general data and identifying symptomatology and mental disorders. The characteristics of the selected document content were statistically analysed.

### Checklist for assessing guides

A new questionnaire for assessing the content of the selected document was designed to identify common patterns (key subjects for decision makers and their links). Questions were selected following the guidelines from the WHO, APA, UN, Centers for Disease Control and Prevention and, finally, MH Europe. The items (39) were initially identified by the authors and a panel of experts from the PSICOST scientific group (Table 1) and organized into three domains: 1) COVID-19 general information, 2) MH strategies and 3) MH recommendations. Domains 2 and 3 distinguished 1) MH topics, e.g., psychological tips and anxiety, from 2) MH-related topics, e.g., people with disabilities and healthcare workers.

**Table 1:** Checklist for assessing the selected guides

Item	Question: Does the document … (answer: Yes or No)	Cod 1^a^	Cod 2^b^
1	*… include information related to the latest information on COVID-19?*	1	
2	*… include information on the strategies designed by the government in response to the pandemic …*)?	1	
3	*… include information on the latest news about the global response to the COVID-19 outbreak?*	1	
4	*… include psychological tips for maintaining good MH and coping with COVID-19?*	2	1
6	*… describe some psychological skills to help people cope with anxiety and worry about COVID-19?*	2	1
8	*… promote social connection at home?*	2	1
26	*… include information on how to support a loved one who is very anxious about COVID-19?*	2	1
27	*… include information on how to manage stress while people await test results?*	2	1
28	*… include information on how to manage stress if people test positive?*	2	1
29	*… include information on stigma and how to reduce it?*	2	1
32	*… include information on how to manage stress and anxiety?*	2	1
39	*… include links for older adults related to any symptoms or mental disorders?*	2	1
5	*… include information on how to maintain a healthy lifestyle?*	2	2
25	*… include special mention of people with disabilities?*	2	2
30	*… include information for healthcare workers?*	2	2
31	*… include information on how to support health care workers?*	2	2
33	*… develop a strategy for identifying healthcare staff needs as a result of the coronavirus pandemic?*	2	2
34	*… include information for domestic violence victims?*	2	2
35	*… include information for caregivers?*	2	2
36	*… include information on financial support for businesses/people affected by COVID-19?*	2	2
37	*… provide advice on medication access during the COVID-19 pandemic?*	2	2
38	*… consider working at home?*	2	2
7	*… provide emotional support, such as conversations for sharing tips online?*	3	1
9	*… describe how to access MH services?*	3	1
10	*… provide phone or online MH services?*	3	1
11	*… offer an online psychological assessment?*	3	1
12	*… provide feedback on the psychological assessment results?*	3	1
13	*… provide MH treatment/intervention alternatives?*	3	1
14	*… provide telephone or online contact with the general practitioner?*	3	1
15	*… provide telephone or online contact with the psychologist?*	3	1
16	*… provide telephone or online contact with another MH professional?*	3	1
17	*… provide an online community forum?*	3	1
18	*… provide suicide and crisis support?*	3	1
21	*… provide steps for understanding children’s feelings?*	3	1
19	*… include information for parents?*	3	2
20	*… include information on how to explain the coronavirus to children?*	3	2
22	*… provide alternatives to older adults to stay connected online?*	3	2
23	*… help in establishing online and learning digital literacy skills?*	3	2
24	*… include guidelines for COVID-19 outbreaks in residential care facilities (for people with physical and MH disabilities, other community-based health facilities,* e.g.*, drug and alcohol services, community MH)?*	3	2

^a^Cod 1: (1) COVID-19 general information, (2) MH strategy and (3) MH recommendation

^b^Cod 2: (1) MH topics (e.g., symptoms, diseases) and (2) MH-related topics (e.g., physical health)

### Indicator groups (variable sets)

The questionnaire items (Table 1) were classified into seven indicator groups (IG) or variable sets.

1. Mental symptoms (IG1). Independent variables: *loneliness*, *sleeping problems*, *bereavement* and *depression*. Non-independent variable (see cluster analysis in this section): *anxiety*. Non-discriminatory variable: *stress*. A non-discriminatory variable has the same value (YES) in all documents and therefore does not provide any information.
2. Mental disorders (IG2). Independent variables: *schizophrenia*, *bipolar*, *depression*, *substance use disorder*, *eating disorder* and *obsessive-compulsive disorder*. Non-independent variables: *anxiety*, *chronic pain* and *dermatillomania*.
3. COVID-19 information (IG3). Independent variable: *Question 1* (Q1). Non-independent variables: *Q2* and *Q3*.
4. MH strategies and MH topics (IG4). Independent variables: *Q29, Q26, Q27* and *Q39*. Non-independent variables: *Q28* and *Q32*. Non-discriminatory variables: *Q4, Q6* and *Q8*.
5. MH strategies and MH-related topics (IG5). Independent: *Q5, Q25, Q30, Q34* and *Q35*. Non-independent: *Q31, Q33, Q36, Q37* and *Q38*.
6. MH recommendations and MH topics (IG6). Independent: *Q9, Q10, Q11* and *Q14*. Non-independent: *Q12, Q13, Q15, Q16, Q17* and *Q21*. Non-discriminatory: *Q7*.
7. MH recommendations and MH-related topics (IG7). Independent: *Q19, Q22, Q23* and *Q24*. Non-Independent: *Q20*.

### Data collection

According to the structure of the questionnaire, CGA and NA independently extracted data (values for the variables, mostly binary 1: YES, the item has been found and 0: NO, the item is not included in the document) from the included studies. If discrepancies between authors were found, LSC made the final decision. The information extracted was related to the country of publication, type of document, symptomatology, mental disorders, COVID-19 information, MH strategies and MH recommendations.

### Cluster analysis

Pearson’s chi-squared test was used to determine the variable independence (significance level 0.05). Symmetric and directional measures provided additional information. Independent variables were used to conduct a two-step cluster analysis (all variables were binary). The distance used was log-likelihood, and the grouping method was the Akaike information criterion.

## Results

### Study selection

**Fig. 1:**
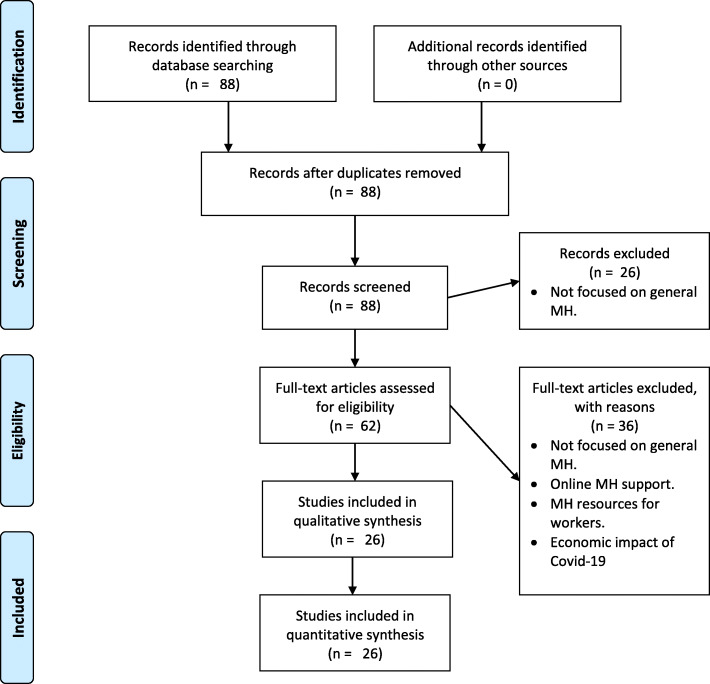
Flowchart and results. From Moher et al., 2009 [46]. Copyright 2009 by Moher et al.

The search strategy identified 88 documents published as of April 15th, 2020, as the most relevant documents at the beginning of the pandemic. No duplicates were found (Fig. 1). In the eligibility phase, 26 documents fulfilled the inclusion criteria (Fig. 1). There was a strong agreement between CGA and NA (*kappa value* = 0.787, *p* = 0.000; *ICC* = 0.881, *p* = 0.000).

### Study characteristics

The general population was the target for almost all the documents (99.06%, Table 2), and the predominant format was a report (73.1%). The documents with more positive “YES” answers were 1 and 13 (85.19%); documents 5 and 11 had a lower rate (25.93%). Documents 1 and 13 were the most complete documents (Table 3) and documents 5 and 11 were the most specific.

**Table 2:** Documents included in the review

Code	Authors (year)	Country^a^	Target population^b^	Document type^c^
1	Australian Government [50]	1	1	2
2	Centre for Addiction and Mental Health [51]	2	1	2
3	Ireland’s Health Services [52]	8	1	2
4	Mental Health Ireland [53]	8	1	2
5	Gobierno de México [54]	10	1	2
6	Mental Health America [55]	16	1	2
7	Centers for Disease Control and Prevention [56]	16	1	2
8	MindHK [57]	3	1	1
9	Centre for Health Protection [58]	3	1	2
10	New Zealand Government [59]	11	1	2
11	Spanish Society of Psychiatry [60]	14	1	1
12	Psychology General Council of Spain [61]	14	1	2
13	Gov.UK (United Kingdom Government) [62]	4	1	2
14	Mental Health Foundation [63]	4	1	2
15	Government of Canada [64]	2	1	2
16	Nidirect Government Services [65]	8	1	2
17	Santépsy.ch [66]	6	1	2
18	Confédération suisse [67]	15	1	2
19	Psychografimata [68]	7	1	2
20	Official College of Psychology of Catalonia [69]	14	1	1
21	Il post [70]	9	1	2
22	MIELI ry [71]	5	1	2
23	Ordem dos psicologos [72]	13	2	1
24	Beyond Blue [73]	1	1	2
25	Australian Psychological Society [74]	1	1	1
26	Mental Health Commission of Canada [75]	2	1	2

^a^Countries: (1) Australia, (2) Canada, (3) China: Hong Kong, (4) England, (5) Finland, (6) Germany, Switzerland and France; CORAASP and CLASS, (7) Greece, (8) Ireland, (9) Italy, (10) Mexico, (11) New Zealand, (12) Portugal, (13) Scotland, (14) Spain, (15) Switzerland and (16) United States of America

^b^Target population: (1) General population and (2) Older adults

^c^Document type: (1) Report and (2) Web page

**Table 3:** Number of positive (YES) answers in percentage (%) per document and indicator group (IG)

Code	IG1	IG2	IG3	IG4	IG5	IG6	IG7	Total
1	50.00	**88.89**	**100.00**	77.78	80.00	**100.00**	**100.00**	**85.19**
2	83.33	66.67	**100.00**	88.89	80.00	91.67	40.00	79.63
3	**100.00**	77.78	**100.00**	44.44	60.00	83.33	60.00	72.22
4	**100.00**	66.67	**100.00**	77.78	40.00	83.33	60.00	72.22
5	33.33	22.22	0.00	33.33	10.00	33.33	40.00	25.93
6	66.67	66.67	**100.00**	88.89	**100.00**	91.67	60.00	83.33
7	83.33	33.33	**100.00**	**100.00**	90.00	75.00	80.00	77.78
8	**100.00**	11.11	66.67	33.33	20.00	50.00	0.00	37.04
9	50.00	22.22	**100.00**	33.33	20.00	83.33	60.00	48.15
10	50.00	11.11	**100.00**	66.67	90.00	**100.00**	80.00	70.37
11	50.00	33.33	0.00	33.33	30.00	16.67	0.00	25.93
12	83.33	22.22	0.00	66.67	50.00	58.33	40.00	50.00
13	**100.00**	44.44	**100.00**	**100.00**	90.00	**100.00**	60.00	**85.19**
14	83.33	77.78	**100.00**	66.67	70.00	83.33	60.00	75.93
15	83.33	22.22	**100.00**	55.56	60.00	75.00	20.00	57.41
16	83.33	22.22	**100.00**	44.44	80.00	83.33	80.00	66.67
17	50.00	66.67	**100.00**	44.44	70.00	66.67	40.00	61.11
18	66.67	66.67	**100.00**	44.44	70.00	66.67	40.00	62.96
19	33.33	11.11	66.67	55.56	60.00	50.00	40.00	44.44
20	66.67	11.11	**100.00**	66.67	60.00	66.67	40.00	55.56
21	66.67	22.22	**100.00**	33.33	40.00	75.00	40.00	50.00
22	50.00	66.67	**100.00**	55.56	60.00	75.00	80.00	66.67
23	66.67	22.22	**100.00**	33.33	20.00	41.67	20.00	37.04
24	**100.00**	22.22	**100.00**	**100.00**	90.00	83.33	80.00	79.63
25	83.33	77.78	**100.00**	33.33	60.00	66.67	**100.00**	68.52
26	83.33	55.56	**100.00**	55.56	70.00	75.00	40.00	66.67

*Note.* Bold guides with higher percentages and shadowed guides with lower percentages

### Results of the cluster analysis

The cohesion and separation profile was excellent (greater than 0.5). No outlier document was found (Table 4). ANOVA showed significant results.

**Table 4:** Cluster analysis results for each indicator group (IG)

Indicator group (IG)	Number of observations					
	Cluster 1	Cluster 2	Cluster 3	Cluster 4	Cluster 5	Cluster 6
IG1 Mental symptoms	7	4	8	7		
IG2 Mental disorders	9	17				
IG3 COVID-19 information	23	3				
IG4 MH strategies & MH topics	8	6	6	6		
IG5 MH strategies & MH-related topics	3	6	5	5	4	3
IG6 MH recommendations & MH topics	6	7	3	10		
IG7 MH recommendations & MH-related topics	4	8	8	3	3	

### Mental symptoms (indicator group 1, IG1)

For IG1 (mental symptoms), almost 100% of the documents had information about *stress* and *anxiety*. *Depression* and *loneliness* were also widely covered (76.92 and 69.23% of the selected documents), and *sleeping problems* and *bereavement* were less frequently referenced (53.85 and 34.62%). Cluster 1 emphasized *anxiety* and *depression*; cluster 2 highlighted *loneliness*, *anxiety* and *stress*; cluster 3 added to the previous *depression*; cluster 4 focused on *sleeping problems*, *stress*, *bereavement* and *depression*.

### Mental disorders (indicator group 2, IG2)

In IG2 (mental disorders), *anxiety* was the most highlighted disorder (96.15%), followed by *depression* and *substance use* (73.08 and 69.23%). The second most common disorders were *eating disorders*, *bipolar* and *obsessive-compulsive* disorders (42.31, 30.77 and 30.77%, respectively). Finally, *somatoform* disorders were less relevant (7.69%). Cluster 1 highlights *schizophrenia*, *anxiety*, *depression*, *substance use* and *eating disorders*; and cluster 2 emphasizes *anxiety*, *depression* and *substance use*.

### COVID-19 information (indicator group 3, IG3)

All the questions had a similar weight [80, 90%] in IG3 (COVID-19 information), and almost all the guides provided relevant information about COVID-19 (79.3%).

### MH strategies & MH topics (indicator group 4, IG4)

**Fig. 2:**
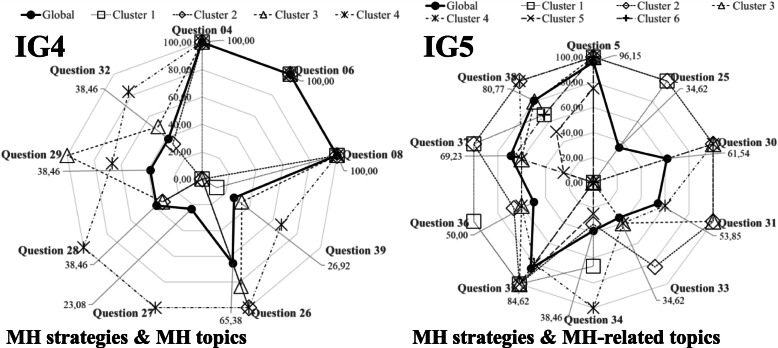
Percentages of positive (YES) answers for IG4 and IG5

For IG4 (MH strategies & MH topics), all the guides include tips for *maintaining good MH*, *describe some psychological skills to help people cope with their anxiety and worry about COVID-19*, and *promote social connection at home*. Globally speaking (global layer, Fig. 2), question 26 (*information on how to support a loved one who is very anxious about COVID-19*) is the most relevant question (65.38% “YES”). It is followed by questions 28 (*including information on how to manage stress in case of positive testing)*, 29 (*how to reduce stigma*) and 32 (*how to manage stress and anxiety*) (38.46% each). Question 27 (*stress management while people are waiting for COVID-19 test results*) is less relevant.

In IG4, Cluster 1 highlighted *maintaining good MH* (*Q4*), *descriptions of psychological skills to help people with anxiety and worries* (*Q6*) and *promotion of social connection at home* (*Q8*). Cluster 2, in addition to the previous clusters, emphasized *Q26* again. Cluster 3 also focused on *reducing stigma* (*Q19*) and *Q26*. Finally, Cluster 4 emphasized *Q26, including information on how to manage stress while people wait for results* (*Q27*) and *how to manage stress when they have a positive test* (*Q28*) (Fig. 2).

### MH strategies & MH-related topics (indicator group 5, IG5)

IG5 shows a global pattern (global layer, Fig. 2) dominated by *including information on how to maintain a healthy lifestyle* (*Q5*) and *including information for caregivers* (*Q35*). Less relevant were *the inclusion of information for healthcare workers* (*Q30*), *how to support them* (*Q31*), *developing advice on medication access* (*Q37*), and *working at home* (*Q38*). Cluster 1 highlighted *information on how to maintain a healthy lifestyl*e (*Q5*), *including special mentions of people with disabilities* (*Q25*), *including information for caregivers* (*Q35*), *including information on financial support* (*Q36*) and *developing advice for medication access* (*Q37*). Cluster 2 focused on *Q5* and *Q25, including information and support for healthcare workers* (*Q30*), *including information on how to support health care workers Q31*, *Q35*, and *Q37* and *working at home* (*Q38*). Cluster 3 highlighted *Q5*, *Q30*, *Q31* and *Q35*. Cluster 4 emphasized *Q5*, *Q25*, *information for people suffering from domestic violence* (*Q34*) and *Q38.* Clusters 5 and 6 had only one relevant question each, *Q5* and *Q35,* respectively (Fig. 2).

### MH recommendations & MH topics (indicator group 6, IG6)

The global profile of IG6 highlighted all the questions (from 76.92 to 100%), except: *offer an online psychological assessment?* (*Q11*, 23.08%), *provide feedback on the psychological assessment results* (*Q12*, 19.23%) and *provide telephone or online contact with the general practitioner* (*Q14*, 38.46%). Cluster 1 had the same pattern, but *Q14* was less important. For Cluster 2, *Q11* and *Q12* were not relevant. Cluster 3 highlighted *providing emotional support* (*Q7*) and *providing a community forum* (*Q17*). Cluster 4 emphasized *Q7* and *describes how to access MH services* (*Q9*).

### MH recommendations & MH-related topics (indicator group 7, IG7)

Globally speaking, IG7 highlighted *include information for parents* (*Q19*, 88.46%), *how to explain the coronavirus to children* (*Q20*, 76.92%) and *providing alternatives for older adults to be connected* (*Q22*, 61.54%). Cluster 1 emphasized all the questions except *including guidelines for COVID-19 outbreaks in residential care facilities* (*Q24*). In Cluster 2, *Q19* was the most relevant. Cluster 3 focused on *Q19* and *Q22*. Cluster 4 was dominated by questions *Q19, Q20, Q22* and *Q24*. In Cluster 5, most answers were “NO”, except for *Q22*.

### Linked questions in the documents

Some of the questions were linked in the selected documents, which means that when one question appeared, the other question also appeared (Yes & Yes answers). In IG1 (mental symptoms), most of the guides linked *loneliness* and *depression* (54% of the documents), *anxiety* and *stress* (96%) and *anxiety* and *sleeping problems* (50%) symptoms, and the former two relationships were very strong. This was not the case for *depression* and *bereavement* (27%). The larger cluster (Cluster 3) showed a perfect link between *loneliness* and *depression* and *anxiety* and *stress*, with the relationship between *anxiety* and *sleeping problems* also being strong.

In IG2 (mental disorders), there was a strong link between *anxiety* and *substance use* (65%) and *anxiety* and *depression* (69%), but *anxiety* and *eating disorders* and *anxiety* and *obsessive-compulsive disorder* had lower relevance (42 and 31%). Cluster 2 confirmed the previous pattern, but both the *anxiety* and *substance use* and *anxiety* and *depression* links were less relevant.

In IG3 (COVID-19 information), very few documents (12%) did not link *… includes information related to the latest information on COVID-19* (*Q1*) and *… includes information on the latest news about the global response to the COVID-19 outbreak* (*Q3*).

**Fig. 3:**
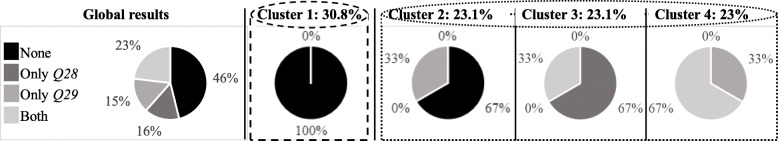
*Q28*-*Q29*

In IG4, most of the selected documents did not *include information on how to manage stress if they have tested positive* (*Q28*) and *include information on stigma and how to reduce it* (*Q29*) (Fig. 3).

**Fig. 4:**
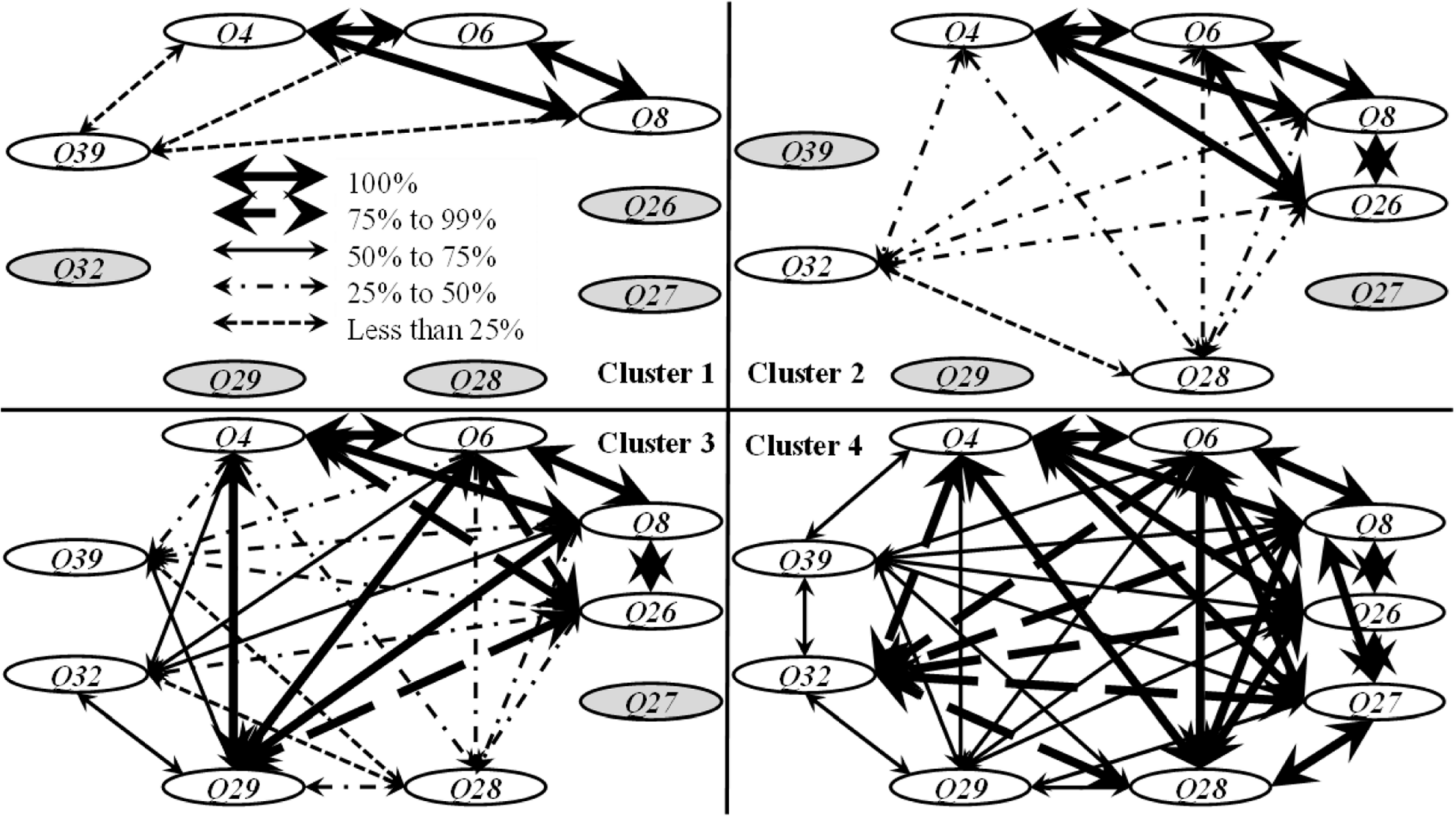
Conceptual maps of the IG4 clusters: evolution of the pattern. Thicker lines represent stronger links

The conceptual maps (Fig. 4) show that the relationships between *including psychological tips for maintaining good mental health and coping with COVID-19* (*Q4*), *describing some psychological skills to help people cope with anxiety and worry about COVID-19* (*Q6*) and *promoting social connection at home* (*Q8*) were very strong. Progressively, strong links between the previous questions and *does the strategy include information on how to support a loved one who is very anxious about COVID-19* (*Q26*) and *Q29* appeared. Finally, the questions *do the strategy include information on how to manage stress while people await test results* (*Q27*), *does the strategy include information on how to manage stress and anxiety* (*Q32*) and *are there any links for older people related to any symptoms or mental disorders* (*Q29*) were less linked.

**Fig. 5:**
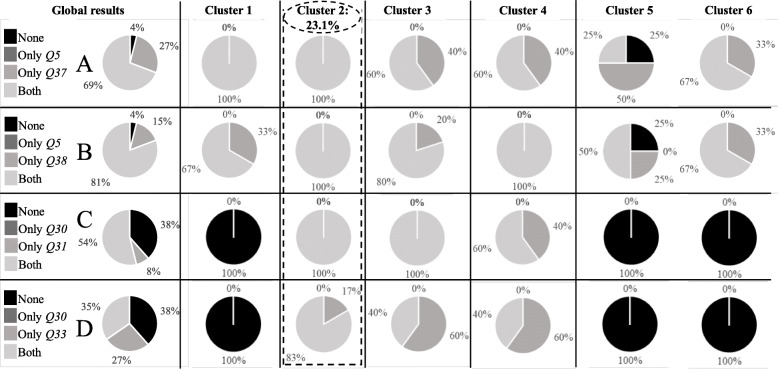
*Q5*-*Q37* (**a**), *Q5*-*Q38* (**b**), *Q30*-*Q31* (**c**) and *Q30*-*Q33* (**d**)

In IG5, there was a strong link between *describing how to maintain a healthy lifestyle.* (*Q5*) and *develop advice on medication access during the COVID-19 pandemic* (*Q37*) (A, Fig. 5) and between *Q5* and *contemplate working at home* (*Q38*) (B). The relationships between *include information for healthcare workers* (*Q30*) and *include information on how to support health care workers* (*Q31*) (C) and between *Q30* and *develop a strategy for identifying healthcare staff needs as a result of the coronavirus pandemic* (*Q33*) (D) were less relevant. Cluster 2 highlighted Yes & Yes in all the analysed links.

In IG6, most of the documents strongly linked *how to access MH services* (*Q9*) and *provide MH treatment/intervention alternatives* (*Q13*, 84%) as well as *Q9* and *provide telephone or online contact with the psychologist* (*Q15*, 81%). The relationship between *providing any phone or online MH services* (*Q16*) and *providing an online community forum* (*Q17*) was also important (52%).

In IG7 and from a global perspective, there was a strong link (77%) between *including information for parents* (*Q19*) and *including information on how to explain the coronavirus to children* (*Q20*). However, in clusters 2 and 3, *Q19* alone was also relevant. All the documents without *Q19* and *Q20* were concentrated in cluster 5.

## Discussion

To the best of our knowledge, this is the first study to collect empirical background on the main online international strategies and recommendations developed to cope with the psychological impact of COVID-19 at a very early stage of the pandemic. Considering its negative MH effects [76, 77], the existence of common patterns is relevant to a) classify documents from a multidimensional perspective (indicator groups), b) help others develop new reports and guides, c) link key subjects appropriately according to the objectives, and d) adapt new documents to local situations in a coordinated way, assuring the dissemination of trusted information.

It was possible to adapt the PRISMA statements to systematically review online international MH reports and guides. Then, by combining statistical analysis, graphical tools and conceptual maps in document structural analysis, researchers can provide quick and reliable results from online webpages and reports.

The new questionnaire successfully assessed the structure and content of the selected documents. Its structure can be considered the gold standard for reviewing online documents related to MH strategy and recommendations. The new methodological approach surpasses qualitative approaches by using cluster analysis, graphical tools and conceptual maps. Selected documents were classified by 1) their degree of complexity, 2) their degree of specificity by grouping the variables and 3) the purpose or objectives to be met (strategic, recommendations, MH topics and MH-related topics).

The common patterns can be studied from a unidimensional or multidimensional perspective. Anxiety and stress symptoms as well as anxiety disorders were included in almost all documents. Comorbid pathologies such as substance use and depression were also frequently incorporated together. Including information about the pandemic characteristics and evolution as well as about the strategies or recommendations designed and implemented to deal with them was also relevant. MH decision makers highlighted the relevance of people being informed by trusted sources because information reduces uncertainty. The selected documents include updated information on international strategies for fighting COVID-19 and how this fight is happening to avoid fear and panic.

All the selected documents emphasize the relevance of available practical tools for managing anxiety and worries about COVID-19 and maintaining social connections to achieve good MH. Most of them also highlight the need to design instruments to help others inside the family. Maintaining a healthy lifestyle and including information on how to take care of others are key subjects developed by the documents.

All countries were concerned about the importance of providing real emotional support. Therefore, a strong e-MH system, including coordinated telecommunications and computer-based tools, is required to meet population needs and distinguish MH users from the general population who need punctual counselling or community support. e-MH allows the screening of pandemic symptoms and mental disorder incidence to prevent new consequences. Knowing how to access MH care is critical in emergency situations. Finally, many documents developed child-adapted information for parents; however, very few had instruments adapted for older adults.

Most of the documents linked loneliness and depression as well as anxiety and stress symptoms, frequently comorbid. Anxiety and substance use as well as anxiety and depression disorders were also related because during the pandemic, people mainly suffer from anxiety, depression, posttraumatic stress and obsessive-compulsive disorder [78, 79].

Surprisingly, most of the documents did not include specific information on how to manage stress for persons who had tested positive or how to reduce the potential stigma of the infection. It is worth highlighting that the inclusion of stigma was related to the document level of complexity; those that concern the identification and reduction of stigma were the most complex ones and have previously included information on, for example, symptoms such as stress and anxiety.

However, many documents linked healthy lifestyle maintenance to advise on medication access (adherence). Both the information availability on medication access and on how to organize this access were considered essential.

Working from home is suddenly new for some professionals. Selected documents provided tips about the management of indispensable contact with supervisors and colleagues to feel supported. Performing safety and hygiene precautions while considering personal finances were essential in this situation [80]. A lack of information about how to manage this type of work could increase anxiety and stress levels.

There is global consensus on the relevance of appropriate information for healthcare workers. Many selected documents link this fact to practical tools for supporting these professionals. Nevertheless, it was less common to find tips for determining the needs of healthcare staff, such as those developed by the Academy of Medical Royal Colleges [81] and the City Mental Health Alliance UK [82].

In the selected documents, planners frequently linked MH services access items to online treatment/intervention alternatives. This fact can be critical for screening and control purposes because of face-to-face constraints.

The main limitation of this review is the increasing number of documents that are being developed in response to the pandemic. This review was performed rapidly at the beginning of the lockdown; therefore, it offers a global vision of the very first reactions of MH planners to address the evolution of the pandemic. Language selection was also a limitation because documents and guidelines were written in the corresponding native languages (Chinese, Korean, etc.) at a very early stage of the pandemic. Only documents published in English, Spanish, French, Italian and Portuguese were included. Finally, selected documents and guidelines were published not only by governments and international organizations but also by mass media, and their reliability is not uniform.

## Conclusions

From a methodological perspective, the PRISMA guidelines can be adapted for carrying out rapid reviews of online documents. This process integrates expert knowledge to adapt the systematic review methodology (search strategy and eligibility criteria) and design questionnaires (key variables and domains) with statistical analysis, graphical tools and conceptual maps to produce useful results for rapid decision making. Cluster analysis identifies independent variables and highlights potential common patterns in the selected indicator groups. Based on these common patterns, conceptual maps show the evolution of document complexity (relationships between variables). Once these critical components (independent variables, common patterns and document complexity) are stated, decision makers can use them to design new context-based strategies and recommendations to support MH users and their families.

From an MH care point of view, this study identifies the most important symptoms, diseases, strategies and recommendations that MH planners selected for designing online documents at a very early stage of the pandemic. In a very complicated and unknown situation, these elements were combined by experts in specific profiles (clusters) from the simplest to the more complex. This finding shows that, from a basic structure, decision makers incorporated new items to improve information coverage. This sequential process is not random but is dominated by stronger links between these elements.

From an MH planning perspective, the results indicate that standard care provision should be progressively adapted to digital care while ensuring acute patient treatments [83]. e-MH is a useful resource in an emergency, but only when staff are specifically trained and receive reliable information to maintain their health and provide better care. To reach these goals, it is necessary to ensure social connections and to reinforce digital infrastructures to reduce gaps, especially in older adult groups [84, 85]. This global health crisis could be an opportunity to reinforce and integrate e-MH in care ecosystems.

It is crucial to develop strategies to avoid the stigmatization of people who have coronavirus, at-risk groups, MH users and healthcare workers. Only the most sophisticated online documents address this issue.

Most of the selected documents include strategies for dealing with children online, but in contrast, the needs of older adults in online spaces are frequently forgotten. Internet literacy skills training for this collective should be improved by including specific links and tools in online documents.

## Data Availability

All data supporting our findings will be shared on request. The documents included in the systematic review are available in the references.

## Abbreviations

MH: Mental health
WHO: World Health Organization
APA: American Psychological Association
UN: United Nations
ICC: Intraclass Correlation Coefficient
IG: Indicator group
